# Supplementary material for: Validation of Takotsubo Syndrome Scoring System
Source: Diagnostics (Basel). 2025 May 23;15(11):1314. doi: 10.3390/diagnostics15111314 (PMC12155197; doi:10.3390/diagnostics15111314)
Supplement: Supplementary file 1 [file diagnostics-15-01314-s001.zip › diagnostics-3602854-supplementary.pdf]

Supplementary Materials

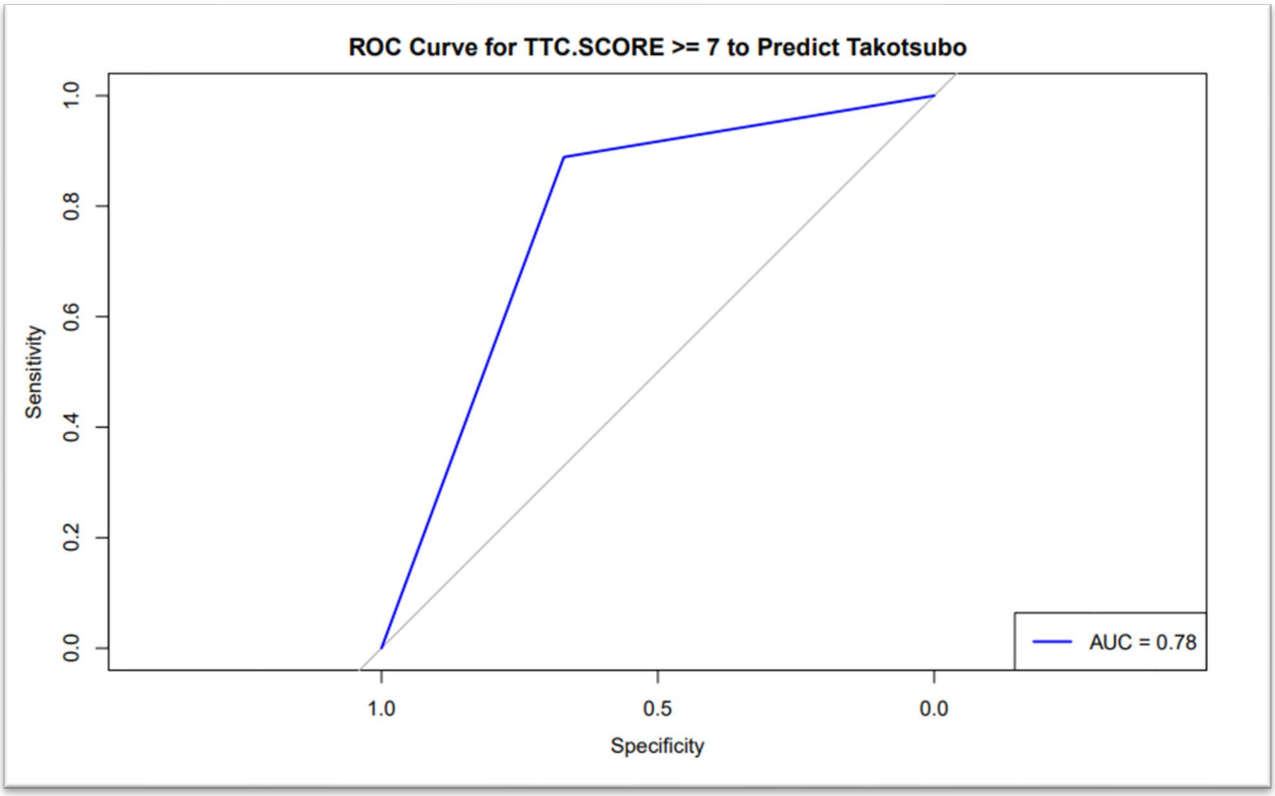

Figure S1: The receiver-operating characteristic (ROC) curve in differentiating Takotsubo syndrome from anterior STEMI; a TS score cutoff ≥ 7 (right) demonstrated moderate diagnostic accuracy, with an area under the curve (AUC) of 0.78, while cutoff ≥ 8 demonstrated excellent diagnostic performance, with an area under the curve (AUC) of 0.84

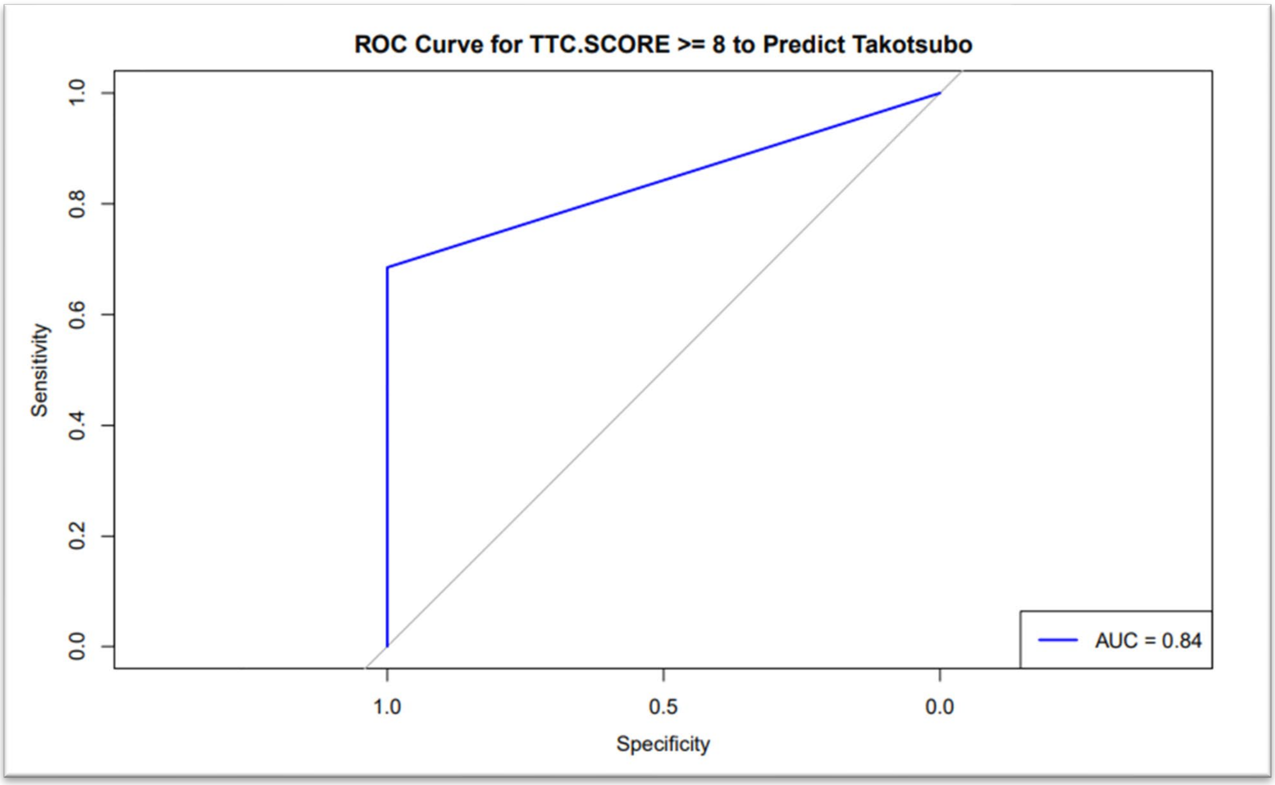

Figure S2: The receiver-operating characteristic (ROC) curve evaluating the TS score (cutoff ≥ 8) for discriminating Takotsubo syndrome (TS group) from all-comer STEMI demonstrated an area under the curve (AUC) of 0.83.

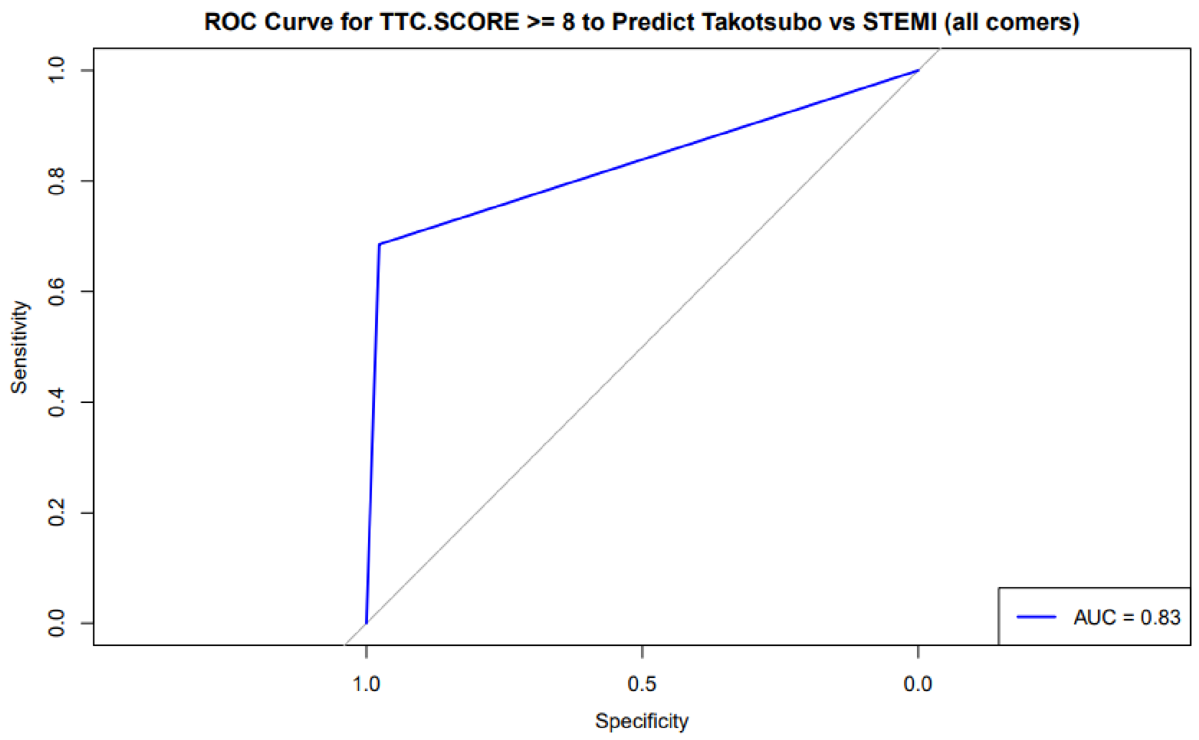

Figure S3: This figure compares the distribution of TS scores between patients with ST-elevation myocardial infarction (STEMI) and Takotsubo syndrome (TS). The TS group demonstrated significantly higher mean scores (7.48 ± 1.63) compared to the STEMI group (5.55 ± 1.32; $p < 0.001$), with minimal overlap in score distributions.

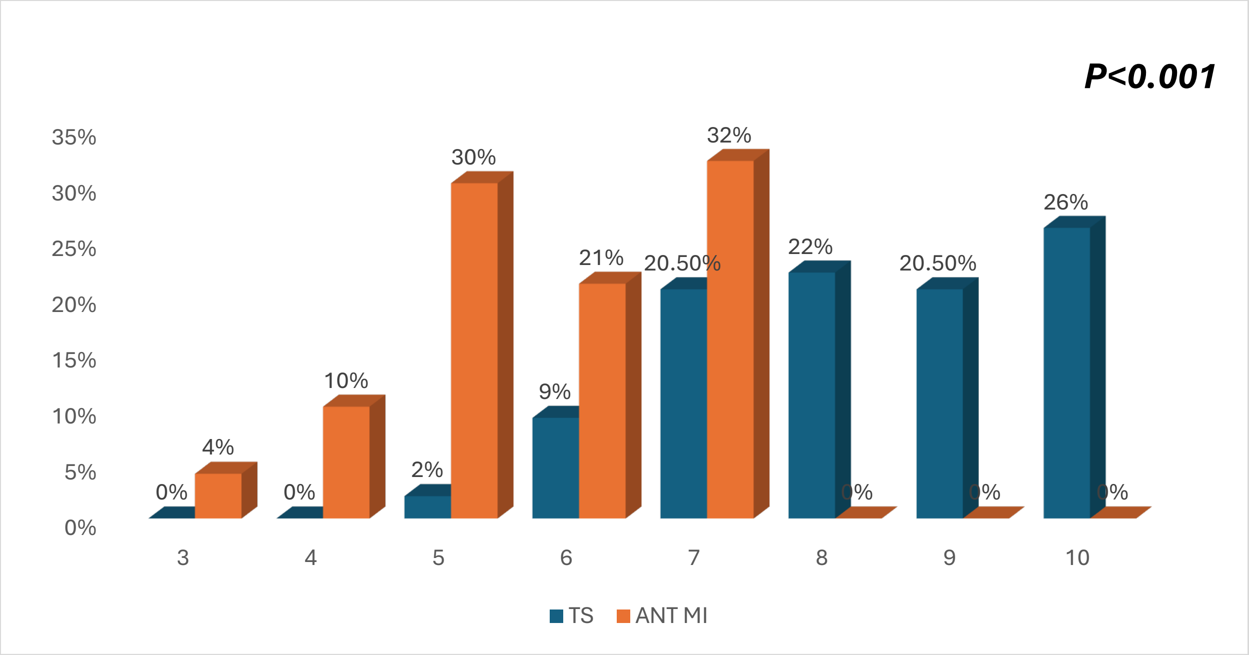

Figure S4: Comparing the TS score between the TS group (blue) and the anterior STEMI group (orange), showing significantly higher scores with the TS group, suggesting the diagnosis of TS.

Table S1: Patient baseline characteristics including baseline characteristics of 3 groups: group 1: females with anterior STEMI; group 2: TS patients; group 3: STEMI all-comers.

	**Female Anterior STEMI (*N* = 97)**	**Takotsubo Syndrome (*N* = 54)**	**All-Comer STEMIs (*N* = 999)**	**Overall (*N* = 1150)**	***p*-Value**
**Age**					
Median (Q1–Q3)	71.0 (64.0–83.0)	68.0 (61.3–78.8)	72.0	70.0 (38–106)	0.041
Mean (SD)	71.2 (12.9)	69.0 (12.1)	72.55 (11.36)	70.97 (12.21)	
**Gender**					
F	97 (100%)	53 (98.1%)	171 (17.1%)	321 (27.91%)	0.001
M	0 (0%)	1 (1.9%)	828 (82.9%)	829 (72.0%)	
**Height**					
Median (Q1–Q3)	1.60 (1.57–1.64)	1.60 (1.55–1.65)	1.7 (1.37–1.9)	1.69	0.001
Mean (SD)	1.61 (0.0595)	1.60 (0.0685)	1.7 (0.09)	1.68 (0.011)	
Missing	2 (2.1%)	0 (0%)	4 (0.4%)	6 (0.52%)	
**Weight**					
Median (Q1–Q3)	68.0 (60.0–77.5)	70.0 (56.3–80.0)	80 (45–160)	79.0	0.001
Mean (SD)	70.0 (13.6)	67.9 (15.7)	80.08 (18.02)	78.3 (17.6)	
Missing	2 (2.1%)	0 (0%)	4 (0.4%)	6 (0.52%)	
**BMI**					
Median (Q1–Q3)	26.4 (24.1–29.8)	26.7 (22.2–31.1)	26.67 (14.86–51.65)	26.7	0.012
Mean (SD)	27.1 (4.54)	26.4 (6.09)	27.63 (5.31)	27.4 (5.3)	
Missing	2 (2.1%)	0 (0%)	4 (0.4%)	6 (0.5%)	
**Ethnicity**					
1	83 (85.6%)	43 (79.6%)	888 (88.9%)	1014 (88.2%)	0.001
2	12 (12.4%)	10 (18.5%)	105 (10.51%)	127 (11.0%)	
3	2 (2.1%)	1 (1.9%)	6 (0.6%)	9 (0.78%)	
**Smoking**					
no	83 (85.6%)	48 (88.9%)	463 (46.3%)	594 (51.65%)	0.001
yes	14 (14.4%)	6 (11.1%)	536 (53.7%)	556 (48.34%)	
**Stressful event**					
no	95 (97.9%)	17 (31.5%)	916 (91.7%)	1028 (89.39%)	<0.001
yes	2 (2.1%)	37 (68.5%)	83 (8.3%)	122 (10.61%)	
**DM**					
no	55 (56.7%)	43 (79.6%)	335 (33.5%)	433 (37.65%)	0.00001
yes	42 (43.3%)	11 (20.4%)	664 (66.5%)	717 (62.35%)	
**LVEF < 40%**					
no	31 (32.0%)	14 (25.9%)	648 (64.9%)	693 (60.26%)	0.001
yes	66 (68.0%)	40 (74.1%)	350 (35.0%)	456 (39.65%)	
**Positive troponin on admission**					
no	3 (3.1%)	0 (0%)	66 (6.6%)	69 (6.0%)	0.023

yes	94 (96.9%)	54 (100%)	933 (93.4%)	1081 (94.0%)	
**TS SCORE**					
Median (Q1–Q3)	6.00 (5.00–7.00)	8.00 (7.00–9.75)	3.83	4.00	<0.001
Mean (SD)	5.7 (1.16)	8.3 (1.39)	3.83 (1.53)	3.92 (1.73)	
